# O−O Bond Formation and Liberation of Dioxygen Mediated by N_5_‐Coordinate Non‐Heme Iron(IV) Complexes

**DOI:** 10.1002/anie.201903902

**Published:** 2019-08-13

**Authors:** Nicole Kroll, Ina Speckmann, Marc Schoknecht, Jana Gülzow, Marek Diekmann, Johannes Pfrommer, Anika Stritt, Maria Schlangen, Andreas Grohmann, Gerald Hörner

**Affiliations:** ^1^ Institut für Chemie Technische Universität Berlin Straße des 17. Juni 135 10623 Berlin Germany; ^2^ Permanent address: Institut für Anorganische Chemie IV Universität Bayreuth Universitätsstraße 30, NW I 95540 Bayreuth Germany

**Keywords:** bioinorganic chemistry, iron, nitrogen ligands, O−O activation, oxo ligands

## Abstract

Formation of the O−O bond is considered the critical step in oxidative water cleavage to produce dioxygen. High‐valent metal complexes with terminal oxo (oxido) ligands are commonly regarded as instrumental for oxygen evolution, but direct experimental evidence is lacking. Herein, we describe the formation of the O−O bond in solution, from non‐heme, N_5_‐coordinate oxoiron(IV) species. Oxygen evolution from oxoiron(IV) is instantaneous once *meta*‐chloroperbenzoic acid is administered in excess. Oxygen‐isotope labeling reveals two sources of dioxygen, pointing to mechanistic branching between HAT (hydrogen atom transfer)‐initiated free‐radical pathways of the peroxides, which are typical of catalase‐like reactivity, and iron‐borne O−O coupling, which is unprecedented for non‐heme/peroxide systems. Interpretation in terms of [Fe^IV^(O)] and [Fe^V^(O)] being the resting and active principles of the O−O coupling, respectively, concurs with fundamental mechanistic ideas of (electro‐) chemical O−O coupling in water oxidation catalysis (WOC), indicating that central mechanistic motifs of WOC can be mimicked in a catalase/peroxidase setting.

Efficient water oxidation catalysis (WOC) is one of the major challenges in the context of future‐oriented energy management schemes. Catalytic water oxidation is a demanding task, owing to its energetic uphill character and the requirement for a coupled multielectron/multiproton shuttle (4 H^+^/4 e^−^) to prevent the formation of hazardous reactive‐oxygen species (ROS). Two types of reagent hold particular promise here: metal‐oxide‐based heterogeneous (electro)catalysts^1^, ^2^, ^3^ and low‐molecular‐weight transition‐metal complexes (typically of Ru; Co, Fe; Ir), which operate in homogeneous solution.^4^, ^5^, ^6^, ^7^, ^8^, ^9^

As for the latter, a number of functional models are now known for the enzyme‐complex‐appended {Mn_4_Ca} cluster, which is the active site of the oxygen‐evolving complex (OEC) in biological photosystem II. Models based on ruthenium are the most numerous; they show robust and efficient oxidative water turnover, have large turnover numbers TON, and use positive electrode potentials or highly oxidizing additives (e.g., cerium(IV) ammonium nitrate).^10^, ^11^ Less numerous to date are models based on 3d metals (Co, Fe).^12^ This is bound to change, however; iron in particular is readily available (Fe being the second most abundant metal in the earth's crust), and there are few, if any, concerns in terms of element toxicity.

Aside from these advantages, current interest in dioxygen‐related iron coordination chemistry has been further fuelled by the following: While metal‐mediated oxygen–oxygen bond formation is generally agreed to be the critical step in both biological photosynthesis and model complex‐based WOC, examples for iron‐mediated O−O bond formation are still rare.^10^, ^13^, ^14^, ^15^, ^16^ High‐valent oxo–iron complexes are invoked as critical intermediates en route to O_2_ liberation—with oxoiron(IV) as the “resting state” and oxoiron(V) as the “active state” of water oxidation, respectively.^17^, ^18^, ^19^ As of yet, however, few details are known regarding the chemical nature of the O−O bond coupling step, and the molecular species involved.

In the following, we report an unprecedented case of efficient O−O bond formation and liberation of dioxygen, mediated by an N_5_‐ligated non‐heme oxoiron(IV) complex in the presence of excess *meta*‐chloroperbenzoic acid (*m*CPBA). We employed the Fe^IV^(O) complex of ligand **L** (**L**=*N* ^1^,*N* ^3^,2‐trimethyl‐2‐(pyridin‐2‐yl)‐*N* ^1^,*N* ^3^‐bis(pyridin‐2‐ylmethyl)propane‐1,3‐diamine; see Scheme 1) and the complex of well‐established **Bn‐TPEN** (*N*‐benzyl‐*N*,*N*′,*N*′‐tris(2‐pyridylmethyl)ethane‐1,2‐diamine).^20^ By a combination of headspace gas analysis and in situ electrochemistry, [Fe^IV^(**L**)(O)]^2+^ has been unambiguously shown to produce dioxygen as a reaction product under the prevailing conditions. We suggest an oxoferryl‐based mechanism, founded on ^16/18^O isotope‐labeling experiments coupled with MS detection.

**Scheme 1:**
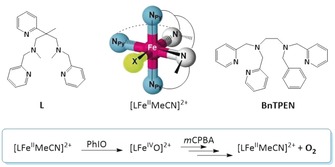
Top: Structures of the pentadentate N_5_ podands **Bn‐TPEN** and **L** and the iron(II) complex, which has dissociable MeCN at the sixth coordination site (**X**). Bottom: Phenomenology of oxoiron(IV) formation and decay as described here.

We had reported on the coordination chemistry and spin state preferences of the pentadentate ligand **L** 21 (its improved synthesis, which gives faster access to **L** in higher yield, is detailed in the Supporting Information, along with additional XRD data of [Fe^II^(**L**)(OTf)](OTf)⋅(0.5 Et_2_O); Figure S1). From the distorted octahedral iron(II) precursor [Fe^II^(**L**)(MeCN)]^2+^ (triflate salt), the oxoiron(IV) complex [Fe^IV^(**L**)(O)]^2+^ is accessible in moderate yields (ca. 30–40 %) by reaction with an equimolar amount of *m*CPBA in MeCN solution,^21^ but forms close to quantitatively with *m*CPBA present in excess (we find an optimum for a ratio [Fe^II^(**L**)(MeCN)]^2+^/*m*CPBA=1:5; see Figure S2; optimum yield >85 %). Similar observations have been reported by Que et al. in a topologically related system.^22^ [Fe^IV^(**L**)(O)]^2+^ is identified through its prominent peak in the ESI mass spectrum, which responds in the expected manner to ^16^O/^18^O isotope exchange, upon treatment of the reaction mixture with H_2_^18^O (Figure S3a). The Vis/NIR spectroscopic properties of [Fe^II^(**L**)(O)]^2+^ (*λ*_max_=730 nm; *ϵ*_730nm_=260 m ^−1^ cm^−1^; Figure S3c) in dilute solution are in the range typical of oxoiron(IV) complexes with Fe in a tetragonal coordination environment.^23^ [Fe^II^(**Bn‐TPEN**)(O)]^2+^ is synthesized in MeCN solution in high yield from [Fe^II^(**Bn‐TPEN**)(OTf)](OTf) according to published procedures.^24^, ^25^ Similar to other non‐heme oxoiron(IV) species,^26^ [Fe^IV^(**L**)(O)]^2+^ exhibits moderate reactivity towards hydrogen‐atom donors (see Figure S4), as well as the oxygen‐atom acceptor PPh_3_ (see Figure S5). However, when potent quenchers are absent but an excess of *m*CPBA is present, solutions of [Fe^IV^(**L**)(O)]^2+^ in MeCN or MeCN/water mixtures spontaneously release dioxygen. To our surprise, previously well‐studied [Fe^IV^(**Bn‐TPEN**)(O)]^2+^ likewise supports dioxygen release under the same conditions.

Dioxygen evolution in solutions of [Fe^IV^(**L**)(O)]^2+^ is unambiguous, as shown by a combination of fiber‐optic sensing of dioxygen in the solution headspace (sensor supplied by PreSens, Regensburg, Germany), and Clark‐electrode measurements in the bulk solution; only the latter technique has been used for [Fe^IV^(**Bn‐TPEN**)(O)]^2+^. Blank experiments with all components, carried out in order to exclude potential apparatus leakage, as well as the direct formation of O_2_ from *m*CPBA in the absence of the iron complex, proved all negative. Using the fiber‐optic sensor,^27^ which is operated discontinuously, significant O_2_ evolution is traceable after the addition of *m*CPBA. Reaction of [Fe^II^(**L**)(MeCN)]^2+^ with 10 equiv *m*CPBA in acetonitrile ([Fe^II^(**L**)(MeCN)]^2+^]=11 mm, *V*_solution_=10 mL, *V*_gas phase_=20 mL) gives approximately 50 μmol of O_2_ in the gas phase (Figure S6), whereas the blind tests using only MeCN, or [Fe^II^(**L**)(MeCN)]^2+^ in MeCN, or *m*CPBA in MeCN, show no such behavior, but detect even traces of dioxygen if these are purposely admitted at a later stage. Reasonably assuming the solution phase to be near‐saturated with dioxygen ([O_2_]_max_≈11 mm 28), oxygen formation amounts to ca. 160 μmol; this renders its formation super‐stoichiometric with respect to the iron content (*n*(O_2_)/*n*(Fe)≈1.5:1).

Continuous monitoring of oxygen evolution in solution was performed with a Clark‐type oxygen electrode system^7^, ^29^ (water/MeCN 4:1; [[Fe^II^(**L**)(MeCN)]^2+^]=2 mm; an aqueous solvent is required for electrode function). After addition of *m*CPBA (10 equiv) to the solution of [Fe^II^(**L**)(MeCN)]^2+^, an instantaneous but gradually diminishing increase of the dioxygen concentration is detected over 30 min (Figure 1 a, blue curve). It is emphasized that a stable plateau signal does not indicate ceased O_2_ evolution, but a steady state of electrochemical consumption and sustained iron‐dependent production. A blank test with only *m*CPBA in the solution showed a very slight, if any, increase in the oxygen signal. The initial rate of O_2_ evolution via [Fe^IV^(**L**)(O)]^2+^ is estimated to be 0.2 μmol min^−1^, translating into an (apparent^30^) initial turnover frequency *TOF*_0_≈2.8 h^−1^ in the presence of 10 equiv mCPBA. Both the initial slope and the step height grow in proportion with the amount of *m*CPBA added. Importantly, aged solutions can be re‐activated by iterative administration of *m*CPBA aliquots (Figure S7). Recovery of the initial reactivity indicates efficient recovery of the reactive iron principle. Under identical conditions, [Fe^IV^(**Bn‐TPEN**)(O)]^2+^ likewise supports oxygen evolution (Figure S8). Diminished peak oxygen concentrations and less sustainable O_2_ production indicate an inherently smaller activity due to the subtly altered ligand structure.

**Figure 1:**
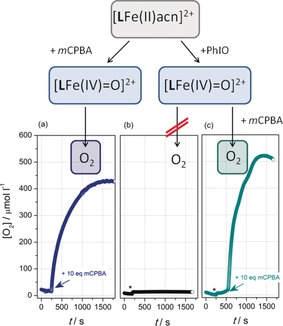
Oxygen evolution (Clark‐electrode system) from the oxoiron(IV) species [Fe^IV^(**L**)(O)]^2+^, as synthesized in MeCN/water (1:4) from the reaction of [Fe^II^(**L**)(MeCN)]^2+^ with a) 10 equiv *m*CPBA, b) 2 equiv PhIO, and c) 2 equiv PhIO followed by 10 equiv *m*CPBA; asterisks denote the addition of PhIO; arrows denote the addition of *m*CPBA.

Intriguingly, solutions of [Fe^IV^(**L**)(O)]^2+^ will produce no O_2_ when [Fe^IV^(**L**)(O)]^2+^ is generated from [Fe^II^(**L**)(MeCN)]^2+^ through reaction with the alternative oxygen‐atom donor PhIO (stoichiometric equivalent or slight excess; Figure 1 b, black curve); the same holds for [Fe^IV^(**Bn‐TPEN**)(O)]^2+^. Under such conditions, irreversible deactivation channels with mono‐exponential decay kinetics prevail which do not involve O_2_ formation (intrinsic lifetime of [Fe^IV^(**L**)(O)]^2+^ at ambient temperature from UV/Vis spectroscopy, *τ*_int_≈130 min; Figure S9); in addition, UV/Vis spectra give no indication of the regeneration of [Fe^II^(**L**)(MeCN)]^2+^ from such samples. The authenticity of PhIO‐derived [Fe^IV^(**L**)(O)]^2+^ was established by means of consistent UV/Vis spectroscopic and mass spectrometric data (Figure S3b,c and Ref. 21).

We thus conclude that O_2_ release is not an intrinsic property of the oxoiron(IV) species; rather, the O−O bond‐forming reaction pathway(s) is/are gated by additives. This view is fully corroborated by experiments decoupling the synthesis of [Fe^IV^(**L**)(O)]^2+^ from O_2_ production. Once more, consistent observations are made in the case of [Fe^IV^(**Bn‐TPEN**)(O)]^2+^. In the first step, [Fe^IV^(**L**)(O)]^2+^ is formed from [Fe^II^(**L**)(MeCN)]^2+^ through reaction with 2 equiv PhIO. Such inactive solutions of [Fe^IV^(**L**)(O)]^2+^ can be activated in a second step and immediately produce significant amounts of O_2_ upon addition of *m*CPBA (Figure 1 c; green curve). Both the initial rate and the turnover frequency (a slight increase is noted; apparent *TOF*_0_≈4.5 h^−1^) are consistent with the observations made in the absence of PhIO. Concomitant with oxygen evolution, UV/Vis spectroscopy reveals a massively enhanced apparent lifetime of [Fe^IV^(**L**)(O)]^2+^ which is dependent on the presence of *m*CPBA; 240 min after addition of *m*CPBA, the concentration of [Fe^IV^(**L**)(O)]^2+^ still amounts to ca. 70 % of the peak concentration and slowly fades on even longer timescale (Figure S10). This finding should be compared with a residual level of <5 % [Fe^IV^(**L**)(O)]^2+^ in the absence of *m*CPBA. We associate the apparent persistence of [Fe^IV^(**L**)(O)]^2+^ with its *m*CPBA‐dependent regeneration from intermediate [Fe^II^(**L**)(MeCN)]^2+^, akin to steady‐state behavior; that is, [Fe^IV^(**L**)(O)]^2+^ is an integral part of a cyclic process which consumes *m*CPBA upon its formation and consumption. As is shown below, [Fe^II^(**L**)(MeCN)]^2+^ finally accumulates in “spent” solutions of [Fe^IV^(**L**)(O)]^2+^, most probably after complete consumption of *m*CPBA (even in very dilute solutions, <0.1 mm, reaction of 1 equiv [Fe^II^(**L**)(MeCN)]^2+^ and 1 equiv *m*CPBA is rapid and complete). Clearly, the observed activation and the persistence are due to the peracid as such; addition of *meta*‐chlorobenzoic acid *m*CBA (10 equiv), which could, in principle, support O−O bond formation as a bridging ligand in a binuclear scenario, neither affects the lifetime of [Fe^IV^(**L**)(O)]^2+^ nor does it support dioxygen release.

Undoubtedly, dioxygen formation occurs in solutions of [Fe^IV^(**L**)(O)]^2+^, as well as [Fe^IV^(**Bn‐TPEN**)(O)]^2+^, with the complexes being the active principles. Any pathways dependent on “free” iron ions can be ruled out as oxygen evolution does not occur in mixtures of iron(II) salts and *m*CPBA. Isotope labeling studies in the presence of ^18^OH_2_ support this conclusion and allow insights into the nature of the O−O coupling step; ion currents *i*_*m*/*z*_ at selected mass/charge ratios are recorded as measures of isotopomer speciation. After treatment of presynthesized [Fe^IV^(**L**)(O)]^2+^ with 10 equiv *m*CPBA in MeCN, MS analysis of the headspace identifies O_2_ and significant amounts of carbon dioxide as gaseous products, irrespective of labeling. By contrast, CO_2_ is absent when [Fe^IV^(**L**)(O)]^2+^ is derived from PhIO and subsequently reacted with *t*‐BuOOH. Thus, the formation of both O_2_ and CO_2_ is triggered by *m*CPBA. Carbon dioxide formation implies formation of significant amounts of elusive RCO_2_^.^ (with *R*=3‐chlorophenyl); such aromatic carboxyl radicals are known to undergo rapid and selective decarboxylation, RCO_2_^.^ → R^.^ + CO_2_.^31^, ^32^ They may derive from parent RCO_3_H via a HAT‐initiated bimolecular sequence or formal loss of a hydroxyl radical OH^.^ (Scheme 2 a,b) or through O−O bond homolysis of iron(III) acylperoxido species (i.e., [Fe^III^(**L**)]O_3_CR]^2+^ → [Fe^IV^(**L**)(O)]^2+^ + RCO_2_^.^).^22^

**Scheme 2:**
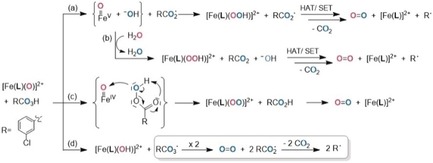
Gated formation of the O−O bond from the reaction of oxoiron(IV) with *m*CPBA; oxygen atoms susceptible to isotope labeling are highlighted in red.

In neat MeCN the measured ratio of dioxygen isotopomer ion currents *i*_32_/*i*_34_≈200:1 matches the isotope distribution expected from natural abundance (32‐O_2_ in Figure 2, left). Insertion of a pre‐equilibration step in the presence of ^18^O‐labeled water (purity: 97 % ^18^O^33^) in the above reaction sequence induces massive shifts in the product ratio. Isotopomer ratios of *i*_32_/*i*_34_≈3:2 (from three iterations; Figure 2, middle) and *i*_32_/*i*_34_≈1:1.1 (from two iterations; Figure 2, right) for [Fe^IV^(**L**)(O)]^2+^ and [Fe^IV^(**Bn‐TPEN**)(O)]^2+^, respectively, indicate substantial yet incomplete ^18^O monolabeling of liberated dioxygen. It is noted that the ^18^O homo‐isotopomer 36‐O_2_, as the doubly labeled product, has practically no existence in experiments with [Fe^IV^(**L**)(O)]^2+^. This observation—taken together with the absence of oxygen evolution from [Fe(**L**)(O)]^2+^ in the absence of *m*CPBA—definitely rules out both standard mechanisms commonly discussed in water oxidation catalysis studies elsewhere:^34^ direct nucleophilic attack of [Fe^IV^(**L**)(^18^O)]^2+^ by ^18^OH_2_, and head‐to‐head radical coupling of two [Fe^IV^(**L**)(^18^O)^2+^ moieties. While there is a significant “oxoiron dimer” feature in the high‐resolution mass spectra of reaction solution samples (Figure S14) at *m*/*z*=1341.2006, which corresponds to a species {[[Fe^IV^(**L**)(O)]^2+^_2_](OTf)_3_}^+^, this must be due to a triflate‐bridged aggregation of [Fe^IV^O] units, which lacks an O−O bond. In actual fact, upon collision‐induced dissociation, the mass‐selected species {[[Fe^IV^(**L**)(O)]^2+^_2_](OTf)_3_}^+^ does not release O_2_ but selectively yields {[Fe^IV^(**L**)(O)](OTf)}^+^ (*m*/*z=*596.1236) under elimination of neutral [Fe^IV^(**L**)(O)](OTf)_2_; additional loss of formaldehyde leads to {[Fe(**L**‐CH_2_)](OTf)}^+^ (*m*/*z=*580.1125; Figure S15). Intriguingly, topologically closely related [Fe^IV^(**Bn‐TPEN**)(O)]^2+^ gives minor but significant contributions of the 36‐O_2_ isotopomer, most probably via nucleophilic water or hydroxide ion (see below) attack, at least in part (Figure 2, right).

**Figure 2:**
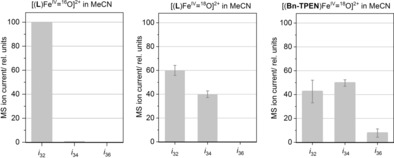
Maximum amplitudes of dioxygen MS ion currents over “dry” MeCN solutions of [Fe^IV^(**L**)(O)]^2+^ (presynthesized via 10 mm [Fe^II^(**L**)(MeCN)]^2+^ + 2 equiv PhIO); left: [Fe^IV^(**L**)(O)]^2+^ in native “dry” MeCN after addition of 10 equiv *m*CPBA, middle: [Fe^IV^(**L**)(O)]^2+^ after labeling with 100 μL ^18^OH_2_ for 30 min and addition of 10 equiv *m*CPBA; right: [Fe(**Bn‐TPEN**)(O)]^2+^ after labeling with 100 μL ^18^OH_2_ for 30 min and addition of 10 equiv *m*CPBA (for ion current vs. time plots, see Figures S11–S13).

The detection of significant amounts of 34‐O_2_ necessarily implies efficient coupling between ^18^O‐labeled iron‐borne oxygen and a ^16^O oxygen atom from another source. This source must be unlabeled *m*CPBA,^35^ as no O_2_ formation is observed in the absence of this reagent. Significant background levels of normal 32‐O_2_ could, in principle, be attributed to slow or incomplete isotope exchange in the species at hand; the residual ^16^OH_2_ content in “dry” MeCN batches used throughout actually reduces the labeling level of ^18^O to ca. 80 %.^33^ In keeping with this, variation of the equilibration time (15 min < *t*_eq_ < 100 min) has no significant effect on the observed product ratios. Indeed, our observed time range covers and exceeds the equilibration times typically necessary for complete ^16^O/^18^O exchange in oxoiron(IV) complexes.^36^ Therefore, we ascribe the major part of trivial 32‐O_2_ formed in solutions of [Fe^IV^(**L**)(O)]^2+^ and *m*CPBA (and *tert*‐butyl hydroperoxide, *t*‐BuOOH) to the operation of free‐radical pathways (Scheme 2 d). It is well known that organic peroxyl radicals are efficient sources of dioxygen via spontaneous decay of labile polyoxide intermediates (e.g., 2 *t*‐BuOO^.^ → (*t*‐BuOO)_2_ → 2 *t*‐BuO^.^ + O_2_).^37^, ^38^ This pathway, which has been recently studied in some detail for iron complexes of a related pentadentate ligand by Browne, McKenzie, and co‐workers,^39^ must be taken to be relevant in our system, as the oxoiron(IV) complex [Fe^IV^(**L**)(O)]^2+^ is competent in HAT reactions (Figure S4 and Ref. 21). In fact, reaction of *t*‐BuOOH and presynthesized [Fe^IV^(**L**)(O)]^2+^ exclusively yields the trivial isotopomer 32‐O_2_, irrespective of the isotope speciation of added water (Figures S16–18; due to the water content of commercial *t*‐BuOOH (30 wt %), the ^18^OH_2_ level amounts to ca. 50 % in MeCN solution) corroborating a catalatic nonscrambling mechanism.^40^, ^41^ In agreement with the notion of the O−H bond in *t*‐BuOOH being much weaker than that in *m*CPBA (literature data based on *t*‐BuOOH and peracetic acid, MeCO_3_H, suggest a difference in bond dissociation energies Δ*BDE*(O‐H)≈36 kJ mol^−1^),^42^ HAT from *t*‐BuOOH fully outcompetes iron‐complex‐borne reactions;^43^ as a matter of fact, the latter become competitive when *m*CPBA is used.

Owing to its highly electrophilic nature, the oxo ligand in [Fe^IV^O] is generally assumed to be susceptible to nucleophilic attack.^44^ Two plausible pathways of the iron‐borne O−O coupling are shown in Scheme 2. The oxoiron(V) path (a) alludes to ideas as expressed by Costas and others,^17^, ^18^, ^19^, ^45^, ^46^ whereas the concerted O‐atom transfer (c) adopts the mechanistic paradigm of *m*CPBA‐driven olefin epoxidation.^47^, ^48^

The latter concerted pathway invokes essentially simultaneous peroxo O−O bond breaking and O_2_ formation within a cyclic intermediate (Scheme 2 c). Although it shares some similarity with the ideas put forward by Hager et al.,^13^ in order to rationalize the formation of dioxygen in the reaction of ferric heme‐dependent chloroperoxidase with *m*CPBA, we favor the oxoiron(V) pathway for the following reasons: The observed *m*CPBA‐gating of dioxygen formation clearly identifies the oxoiron(IV) species [Fe^IV^(**L**)(O)]^2+^ as a resting state of O_2_ production. The similarity to the conclusions drawn by Costas and Lloret‐Fillol et al. from iron–WOC experiments is obvious. Accordingly, the activation of [Fe^IV^(**L**)(O)]^2+^ in the presence of excess *m*CPBA may involve single‐electron oxidation to yield a (formal) oxoiron(V) species, which can be attacked by the incipient hydroxyl (Scheme 2 a). Alternatively, the residual iron(III) produced in a side reaction of incomplete oxoiron(IV) formation may form oxoiron(V) in a heterolytic cleavage reaction of iron(III) acylperoxido species, [**L**Fe^III^‐O_3_CR]^2+^ → [**L**Fe^V^(O)]^3+^ + RCO_2_^−^.^49^ Such species have been invoked previously as the active agent in iron‐catalyzed electrochemical water oxidation.^50^ In both types of studies, the formation of oxoiron(V) required highly oxidizing conditions, that is, either high concentrations of the strong chemical oxidant Ce^IV^ (*E*_0_ (Ce^III/IV^)=1.70 V vs. NHE^51^) and otherwise harsh conditions (i.e., pH≈1), or very positive electrode potentials (*E*_p,*a*_=1.58 V vs. NHE^50^). Oxoiron(V) being attacked by OH^−^ formed in an outer‐sphere electron transfer (or via rapid proton transfer from labeled bulk water, ^16^OH^−^ + ^18^OH_2_ → ^16^OH_2_ + ^18^OH^−^, Scheme 2 b) would indeed rationalize the occurrence—if minor—of the doubly labeled product, 36‐O_2_, seen in the reactions of [Fe^IV^(**Bn‐TPEN**)(O)]^2+^. The subtle effect of ligand structure on the O_2_ speciation justifies further scrutiny.

This nucleophilic O−O coupling is the microscopic reversal of heterolytic O−O cleavage in iron(III) hydroperoxido species; it has been found in DFT studies on the N‐methyl analogue of [Fe^III^(**Bn‐TPEN**)(OOH)]^2+^ to have a huge driving force.^52^ Nevertheless, it appears unlikely that the mild oxidant *m*CPBA used in our study can efficiently drive the Fe^IV^ → Fe^V^ oxidation step in an outer‐sphere electron transfer reaction (but see Ref. 53). However, concerted inner‐sphere transfer of OH^−^ and of an electron in opposite directions avoids the high energy penalties usually attending charge‐building reactions. It appears plausible to ascribe the formation of monolabeled 34‐O_2_ to this net inner‐sphere transfer of a hydroxyl radical;^54^ it is conceptually complementary to the coupled transfer of a proton and an electron, PCET,^55^ which in the meantime has proven its omnipresence in bioinorganic research.

Irrespective of the actual O−O coupling mechanism, the postulated intermediate hydroperoxido (Scheme 2 a) and peroxido complexes (Scheme 2 c), respectively, are obviously labile under the reaction conditions, so that no accumulation is possible. In the case of the peroxido complex, simple ligand exchange with solvent MeCN provides a favorable exit channel, yielding O_2_ and the precursor complex [Fe^II^(**L**)(MeCN)]^2+^. Indeed, dioxygen and carbon dioxide evolution in solutions of [Fe^IV^(**L**)(O)]^2+^ and *m*CPBA is accompanied by the regeneration of complex [Fe^II^(**L**)(MeCN)]^2+^ (as detected by UV/Vis and ^1^H NMR spectroscopies, see below). The fact that the iron(II) precursor regenerates itself partially upon standing rationalizes the observation that the reactivity of “spent” solutions of [Fe^IV^(**L**)(O)]^2+^ can be restored by administration of additional aliquots of *m*CPBA (vide supra). It is noted, however, that the regeneration of [Fe^II^(**L**)(MeCN)]^2+^ may also be traced to the iron(III) hydroperoxide complex implied in Scheme 2 a,b via an additional one‐electron oxidation or HAT reaction.^56^

Whereas the nature of the oxygen‐liberating iron species is unclear at present, regeneration of [Fe^II^(**L**)(MeCN)]^2+^ is beyond doubt. Notably, the new near‐UV band peaking at *λ*=398 nm, which evolves after complete decay of the oxoiron(IV) intermediate, coincides with the spectral response of the iron(II) precursor [Fe^II^(**L**)(MeCN)]^2+^ (Figure 3 a). Significant absorption at *λ*<320 nm indicates the presence of side products, presumably iron(III) species.^57^ A more conclusive spectroscopic argument comes from time‐dependent ^1^H NMR spectroscopy (Figure 3 b). After addition of *m*CPBA (10 equiv), the widely spread resonances ([Fe^II^(**L**)(MeCN)]^2+^ in MeCN is a spin crossover system with *T*_1/2_≈320 K; [hs]/[ls]≈1:4 at RT^21^) of [Fe^II^(**L**)(MeCN)]^2+^ in d_3_‐MeCN are instantly quenched (NMR spectroscopic studies of oxoiron(IV) species are generally rare)^58^ but are recovered in a slow process, returning to ca. 40 % of the initial integrated intensity after 12 h (higher yields will likely be obtained on an extended timescale, see Figure S19). To the best of our knowledge, the Fe^II^→Fe^IV^→Fe^II^ reversion sequence has only a single precedent in related literature: The iron(II) precursor [Fe^II^(**N4Py**)(MeCN)]^2+^ (**N4Py**: *N*,*N*‐bis(2‐pyridylmethyl)‐*N*‐[bis(2‐pyridyl)methyl]amine) is recovered from aged aqueous solutions of the corresponding oxoiron(IV) species in the presence of excess H_2_O_2_;^59^ of particular note here is the fact that the cited work reports iron‐dependent dioxygen formation prior to precursor recovery, presumably via nonscrambling disproportionation. As no labeling studies have been reported, the mechanistic relatedness of the two systems cannot, however, be judged with certainty.

**Figure 3:**
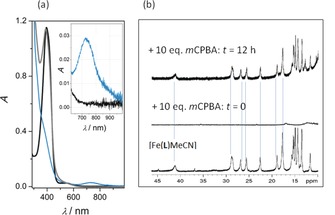
a) UV/Vis/NIR spectral dynamics of [Fe^II^(**L**)(MeCN)]^2+^ (0.14 mm, MeCN, *t*=0; black curve) after addition of 20 equiv *m*CPBA (blue curve: *t*=10 min; gray curve *t*=140 h). b) ^1^H NMR spectroscopic dynamics of [Fe^II^(**L**)(MeCN)]^2+^ (10 mm; d_3_‐MeCN; bottom) directly after addition of 10 equiv *m*CPBA (middle) and after 12 h (top); dashed lines are given to guide the eye.

In the present work, we have reported an unprecedented aspect of non‐heme oxoiron(IV) reactivity: Firstly, our work, which uses non‐heme iron(II) complexes of pentadentate ligands, adds two new examples to the short list of exceptions^50^, ^60^, ^61^ from the “two open *cis*‐sites” rule, which describes a putative structural requirement for an active water oxidation catalyst or, more specifically, for complexes which support metal‐borne O−O bond formation. Oxoiron(IV) complexes of the two N_5_ ligands studied herein do in fact spontaneously produce stoichiometric amounts of dioxygen when the O‐atom‐donor *m*CPBA is present in excess, but are metastable in its absence. The dependence of O_2_ formation on the presence of an excess of *m*CPBA renders oxoiron(IV) a resting state of dioxygen formation. Accordingly, isotope labeling studies reveal a mechanistic branching between nonproductive HAT‐like reactivity and, presumably, OH‐group transfer, with the implicit passing through an oxoiron(V) intermediate. Secondly, the heterocoupling between two different types of activated oxygen species, oxoiron(IV) and a peracid, is established in the present study. While the speciation implied herein probably differs from WOC, the option to study O−O coupling in isolation is expected to be a valuable tool for the scrutiny of the O−O coupling step in WOC, even more so since peroxides have been previously shown to be active principles in WOC.^61^ There are no peculiarities in the structure of the N_5_ ligands **L** and **Bn‐TPEN** with respect to donor speciation and topology, and we are confident that observations similar to ours will be made in the future with other non‐heme systems involving pentacoordinating ligands. The decisive requirement is O_2_‐indifference of the iron(II) precursors (as O_2_ is liberated with concomitant re‐formation of the ferrous complex). This is a property shared by the complexes studied herein, [Fe^II^(**L**)(MeCN)]^2+^ and [Fe^II^(**Bn‐TPEN**)(MeCN)]^2+^.^20^, ^62^ Overall, the O−O bond formation pattern observed in the present work is a unique reversal of the paradigmatic iron‐mediated O−O bond cleavage activity,^63^, ^64^, ^65^ which usually renders non‐heme oxoiron complexes active in H‐atom abstraction^26^, ^66^, ^67^ and oxygen‐atom transfer chemistry.^68^

## Conflict of interest

The authors declare no conflict of interest.

## Supporting information

As a service to our authors and readers, this journal provides supporting information supplied by the authors. Such materials are peer reviewed and may be re‐organized for online delivery, but are not copy‐edited or typeset. Technical support issues arising from supporting information (other than missing files) should be addressed to the authors.

Supplementary
