# Supplementary material for: O−O Bond Formation and Liberation of Dioxygen Mediated by N5‐Coordinate Non‐Heme Iron(IV) Complexes
Source: Angew Chem Int Ed Engl. 2019 Aug 13;58(38):13472–8. doi: 10.1002/anie.201903902 (PMC6772150; doi:10.1002/anie.201903902)
Supplement: Supplementary file 1 — Supplementary [file ANIE-58-13472-s001.pdf]

Angewandte Chemie
Eine Zeitschrift der Gesellschaft Deutscher Chemiker

Supporting Information

O−O Bond Formation and Liberation of Dioxygen Mediated by N_5-Coordinate Non-Heme Iron(IV) Complexes

Nicole Kroll, Ina Speckmann, Marc Schoknecht, Jana Gülzow, Marek Diekmann, Johannes Pfrommer, Anika Stritt, Maria Schlangen, Andreas Grohmann, and Gerald Hörner**

anie_201903902_sm_miscellaneous_information.pdf

Electronic Supporting information

Experimental and Computational Details **S2**

Ligand and Complex Synthesis **S3**

Crystallographic Details **S4**

UV-Vis and MS spectra of $[Fe^{II}(L)(O)]^{2+}$ **S5**

Reactivity of $[Fe^{IV}(L)(O)]^{2+}$ and $[Fe^{IV}(Bn\text{-}TPEN)(O)]^{2+}$ **S8**

Experimental details:

UV/Vis. All measurements were performed with a Varian Cary 50-spectrometer, using 3 ml quartz cuvettes (d = 1cm) at RT. Iron(II) sample solutions were prepared through dilution from concentrated stock solutions kept under N_2. *m*CPBA aliquots were taken from concentrated stock solutions in order to minimize volume changes. After mixing the reactants, the system is allowed to equilibrate for 10 s, before iterative spectral scans are started. Kinetic runs utilized a probe wavelength fixed at λ = 735 nm; absorption was read at time intervals of 30 s.

NMR. ^{1}H-NMR-spectra were recorded with a Bruker Avance II 400 MHz spectrometer. Phenyltrimethylsilane (PTMS, δ = 0.27 ppm) in CD_3CN, filled in a coaxial insert, was used as an external standard. To start the reaction, to a solution of [Fe^{II}(**L**)(OTf](OTf) (21 mM) in CD_3CN (0.7 ml) *m*CPBA (10 eq) was added. The reaction spectra were recorded at time intervals of 30 min for 6h.

XRD analysis. The data were recorded at 150 K with an Agilent SuperNova diffractometer equipped with an Atlas CCD-detector and using graphite-monochromated Cu-K_α radiation (λ = 1.54184 Å). For data collection, the software package CrysAlis CCD and CrysAlis Pro were used. The crystal structure was solved with SHELXS-97, using direct methods, and refined with SHELXL-97 against F_0^2 data by using the full-matrix least squares algorithm. All non-hydrogen atoms were refined anisotropically. All hydrogen atoms were refined isotropically with riding models. CCDC deposition code is given in Table S1.

Mass spectrometry. Mass spectra were recorded using an Orbitrap XL mass spectrometer equipped with an electrospray-ionization (ESI) source. The flow rate of the ESI solution was set to 10µL min^{-1}, the spray voltage to 4.5 kV and the capillary temperature to 275°C.

Oxygen evolution.

Clark-Elektrode (bulk solution). The setup used in this work is a Strathkelvin Instruments 782 Oxygen Meter equipped with a Strathkelvin Instruments 1302 microcathode. A Clark-type electrode is a device capable of measuring traces of dissolved molecular oxygen in aqueous media. It consists of a thin platinum wire (cathode) and an Ag|AgCl type reference electrode in a buffered KCl electrolyte. The electrodes are polarized at a voltage of –0.6 V to –0.8 V relative to the cathode. The electrodes are separated from the reaction mixture by an oxygen permeable polypropylene membrane. 1ml of an oxygen free solution of the compound (2mM, H_2O/MeCN 4:1) was transferred via syringe in an argon filled reaction vessel. The system was allowed to equilibrate and the oxygen signal was monitored for 10 minutes. 1 ml of 10 eq *m*CPBA in H_2O/MeCN (4:1) was added with a syringe to the stirred solution (2x 5 eq: 2x 1 ml, 5x 2 eq: je 0.2 ml). After calibration of the electrode (oxygen free conditions (treatment with Na_2SO_3) versus air-saturated conditions; [O_2] = 287 µmol/L), the current is proportional to the concentration of dissolved oxygen. Blank experiments ((i) H_2O/MeCN + H_2O/MeCN; (ii), H_2O/MeCN + *m*CPBA, (iii) $Fe(OTf)_2$ in H_2O/MeCN + *m*CPBA) all showed negligible signals.

Optometric O_2-Sensor (headspace). Headspace-recording of liberated dioxygen was accomplished by means of the optometric device *Fibox 4 trace* (*PreSens*). Measurements were performed in a Schlenk-tube (total volume 30 ml) under N_2. In a typical experiment, 10 eq *m*CPBA in 2 ml MeCN were added to 8 ml of a stock solution of [Fe(**L**)(OTf)](OTf) (11 mM) in MeCN. Reading of the oxygen

concentration in the solution headspace was performed by means of a calibrated optometric sensor, fixed inside the reaction vessel with silicon glue. Blank experiments ((i) MeCN; (ii) MeCN + complex; (iii) MeCN + *m*CPBA) showed negligible response.

MS isotope labelling study: In a typical experiment the oxo-iron-species was generated by adding 5 equivalents of PhIO to a 10 mM solution of the iron(II)-precursor in dry acetonitrile (1 – 3 ml). Excessive PhIO was filtered after 10 min. For isotopic labelling the resulting solution was stirred for 15 min < t_{eq} < 100 min with $H_2^{18}O$ and then charged with *m*CPBA (10 eq). Gaseous products were transferred to the inlet of an EI-MS (PFEIFFER VACUUM OMNISTAR) by applying continuous helium flow (flow rate 50 ml min^{-1}). Blank experiments ((i) H_2O/MeCN + H_2O/MeCN; (ii), H_2O/MeCN + *m*CPBA, (iii) $Fe(OTf)_2$ in H_2O/MeCN + *m*CPBA, (iv) MeCN/H_2O + PhIO + *m*CPBA, (v) MeCN + *t*-BuOOH, (vi) MeCN + PhIO + *t*-BuOOH, (vii) MeCN/H_2O + PhIO + *t*-BuOOH) all showed negligible signals.

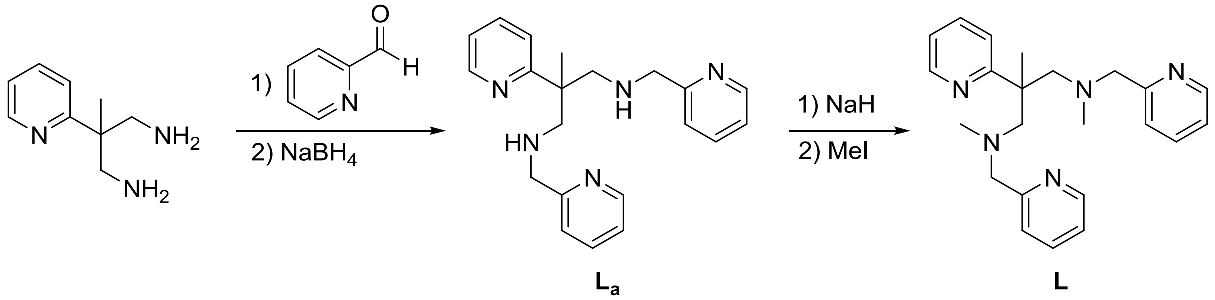

Scheme S1: Synthesis of pentadentate ligand **L**.

Syntheses

2-Methyl-2-(pyridin-2-yl)-*N*^1^,*N*^3^-bis(pyridin-2-ylmethyl)propane-1,3-diamine (L_a)

Picoline aldehyde (5.50 mmol, 0.589 g) is added dropwise to a solution of 2-methyl-2-(pyridin-2-yl)propane-1,3-diamine[1] (2.50 mmol, 0.431 g) in dry MeCN (15 ml) and the mixture left for stirring at room temperature for 6 h. After cooling to 0 °C, $NaBH_4$ (12.5 mmol, 0.473 g) is added and the mixture left for stirring at room temperature for 24 h. The mixture is then made alkaline with saturated Na_2CO_3 and extracted with dichloromethane (3 × 30 ml). The combined organic phases are dried with Na_2SO_4, filtrated and the solvents removed in vacuo, yielding the secondary amine $\mathbf{L_a}$ as a yellow oil (0.80 g; 90 %).

^{1}H-NMR (200 MHz, $CDCl_3$): δ = 8.55-8.50 (m, 2 H, H^{Ar}), 8.49-8.44 (m, 1 H, H^{Ar}), 7.61-7.51 (m, 3 H, H^{Ar}), 7.29-7.22 (m, 3 H, H^{Ar}), 7.12-7.04 (m, 3 H, H^{Ar}), 3.86 (s, 4 H), 2.98 (s, 4 H), 1.39 (s, 3 H) ppm. **HRMS** (ESI(+), MeCN): *m/z* = 348.2182 (calc. 348.2183 for $C_{21}H_{26}N_5$, $[M+H]^+$).

N^1,N^3,2-Trimethyl-2-(pyridin-2-yl)-N^1,N^3-bis(pyridin-2-ylmethyl)propane-1,3-diamine (L)

NaH (60 % in mineral oil, 9.88 mmol, 0.237 g) is suspended in dry DMF (5 ml). At 0 °C a solution of crude $\mathbf{L_a}$ (1.48 mmol, 0.515 g) in dry DMF (15 ml) is added dropwise and the mixture left for stirring at room temperature for 1 h. After cooling to 0 °C, MeI (4.45 mmol, 0.631 g, 0.276 ml) is added

[1] Friedrich, S., Schubart, M., Gade, L. H., Scowen, I. L., Andrew, J., McPartlin, M., *Chem. Ber./Receuil*, 1997, **130**, 1751.

dropwise the mixture left for stirring at room temperature for 2 h. After quenching with saturated NH_4Cl, the resulting solution is extracted with EtOAc (3 $\times$ 30 ml). The combined organic phases are washed with saturated brine. After drying with Na_2SO_4 and filtration, the solvents are removed in vacuo. The residue is triturated with hexanes for 15 min. After decantation the supernatant is then extracted with MeCN. After evaporation of the MeCN phase in vacuo, **L** is obtained as orange-colored oil (0.34 g, 60 %). The recorded analytical data are identical to previously published data of **L**, obtained along an alternative route.[2]

^{1}H-NMR (200 MHz, $CDCl_3$): δ = 8.54-8.49 (m, 1 H, H^{Ar}), 8.44-8.39 (m, 2 H, H^{Ar}), 7.59-7.49 (td, ^3J = 7.6 Hz, ^4J = 1.8 Hz, 3 H, H^{Ar}), 7.42-7.37 (m, 1 H, H^{Ar}), 7.27-7.20 (m, 2 H, H^{Ar}), 7.09-7.04 (m, 2 H, H^{Ar}), 7.01-6.96 (m, 1 H, H^{Ar}), 3.52 (s, 4 H), 3.03 (d, ^2J = 14.3 Hz, 2 H), 2.76 (d, ^2J = 14.3 Hz, 2 H), 1.98 (s, 6 H), 1.53 (s, 3 H) ppm. **HRMS** (ESI(+), MeCN): *m/z* = 376.2503 (calc. 376.2496 für $C_{23}H_{30}N_5$, [*M*+H]$^+$).

[Fe(L)(OTf)](OTf)

After addition of a solution of **L** in MeCN (2 ml) to a suspension of $Fe(OTf)_2$ in MeCN (2 ml), the orange-coloured reaction mixture is stirred for 1 d at room temperature. MeCN is then removed under reduced pressure and the residue triturated with diethyl ether (10 ml) to give a light yellow solid. This is collected by filtration, washed with diethyl ether, and dried *in vacuo*. Analytical data are consistent with previously published data. Isothermal diffusion of diethyl ether into a solution in dichloromethane affords the product as a yellow crystalline material that was suitable for XRD structure elucidation.

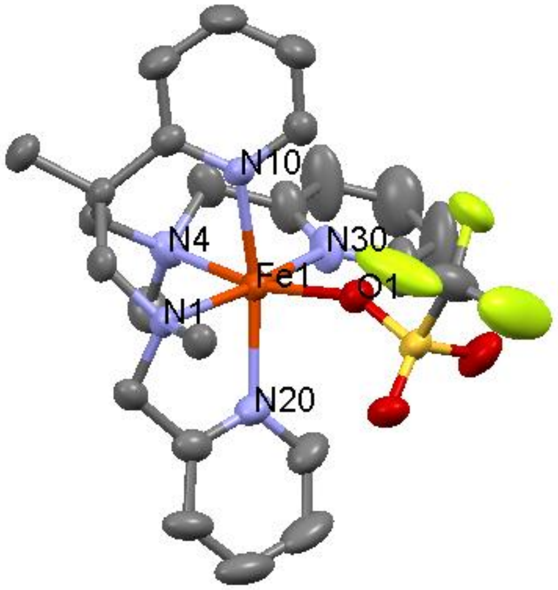

Fig. S1: MERCURY representation of the cation [Fe(**L**)(OTf)]$^+$ in the crystal structure of [Fe(**L**)(OTf)](OTf) · Et_2O; selected bond lengths (Å) and bond angles (°): Fe–N1 2.185(4), Fe–N4 2.237(5), Fe–N10 2.142(4), Fe–N20 2.167(5), Fe–N30 2.190(5), Fe–O1 2.139(4), N4–Fe–O1 164.47(18), N10–Fe–N20 164.88(19), N1–Fe–N30 167.53(19).

[2] Kroll, N., Theilacker, K., Schoknecht, M., Baabe, D., Wiedemann, D., Kaupp, M., Grohmann, A., Hörner, G., *Dalton Trans.* **2015**, 44, 19232.

Table S1: Crystallographic details of [Fe(**L**)(OTf)](OTf) · 0.5 Et_2O

	[Fe(**L**)(OTf)](OTf) · 0.5 Et_2O
CCDC No.	1554991
Radiation	Cu-K_α (λ = 1.54184Å)
Formula	C_{54} H_{68} F_{12} Fe_2 N_{10} O_{13} S_4
M/g · mol^{-1}	1533.12
Crystal dimensions/mm^3	0.27 × 0.25 × 0.10
Crystal description	yellow block
Crystal system	Monoclinic
Space group	$P2_1/c$
a/Å	10.76000 (10)
b/Å	16.43170(10)
c/Å	18.8793(2)
α/°	90
β/°	96.6780(10)
γ/°	90
V/Å^3	3315.31(5)
Z	2
ϱ_{calc}/g · cm^{-3}	1.536
μ/mm^{-1}	5.576
F(000)	1580
T_{min}/T_{max}	0.3144 / 0.6055
Measured reflections/R_σ	20530 / 0.0514
Independent reflections/R_{int}	5982 / 0.0790
ϑ_{min}/°, ϑ_{max}/°	3.58 / 67.50
Data/restraints/parameters	5982 / 259 / 526
R indices ($I > 2\sigma$)	R_1 = 0.0887
	ωR_2 = 0.2075
R indices (all data)	R_1 = 0.0978
	ωR_2 = 0.2140
GoF	S = 1.020
	S' = 1.074
$\Delta\varrho_{fin}$(max/min)/e · Å^{-3}	0.978 / –0.994

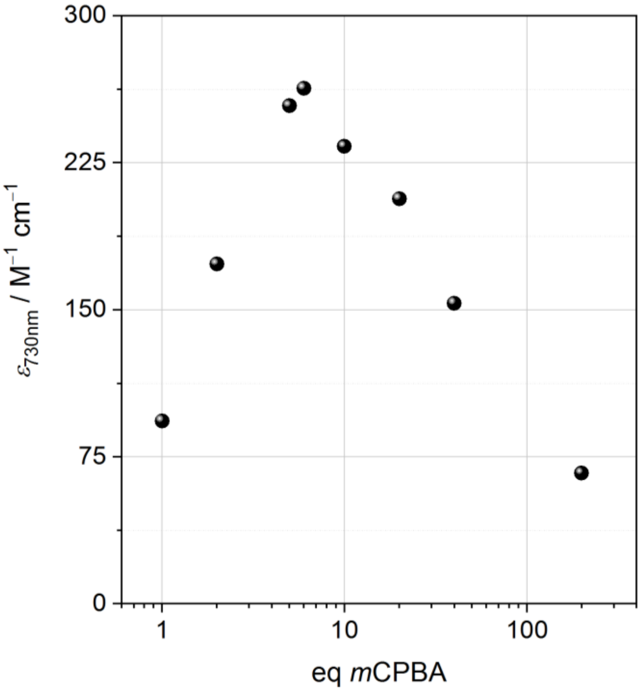

Fig. S2: Yield of oxoiron(IV) complex $[Fe^{IV}(\mathbf{L})(O)]^{2+}$from reaction of $[Fe^{II}(\mathbf{L})MeCN]^{2+}$ ($[[Fe^{II}(\mathbf{L})MeCN]^{2+}]$ = 0.14 mM) in MeCN in dependence of the concentration of *m*CPBA.

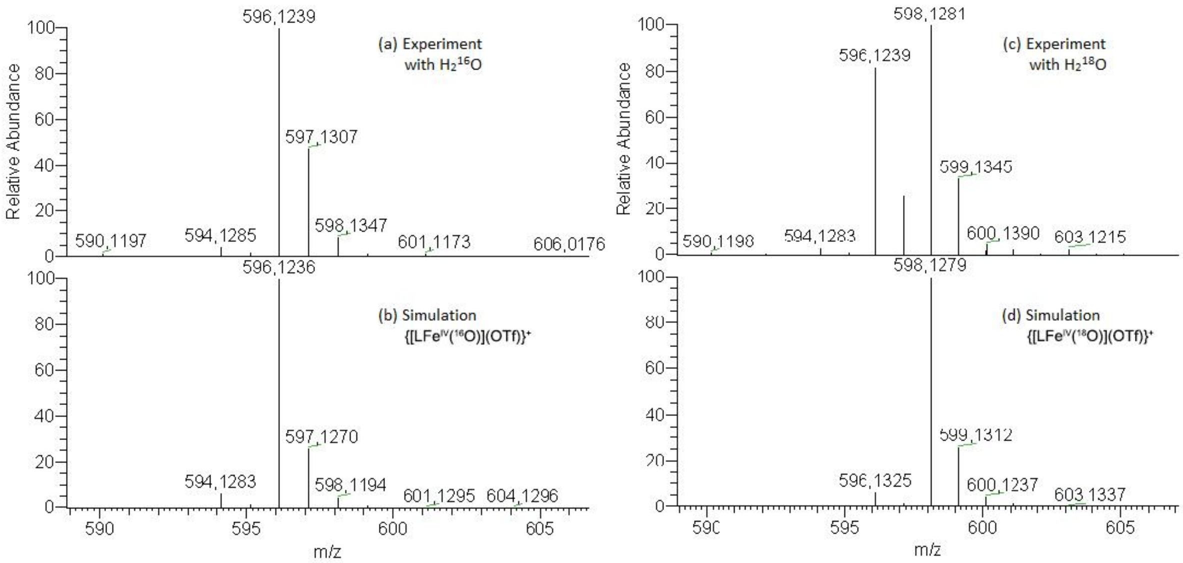

Fig. S3a: ESI-mass spectra of (a) unlabeled and (c) ^{18}O labeled reaction solutions of iron(II) precursor complex $[Fe^{II}(\mathbf{L})MeCN]^{2+}$ and *m*CPBA in MeCN/water mixtures, respectively (isotopic labeling via addition of $^{16}OH_2$ and $^{18}OH_2$, respectively); (b) and (d) show simulations of the experimental data.

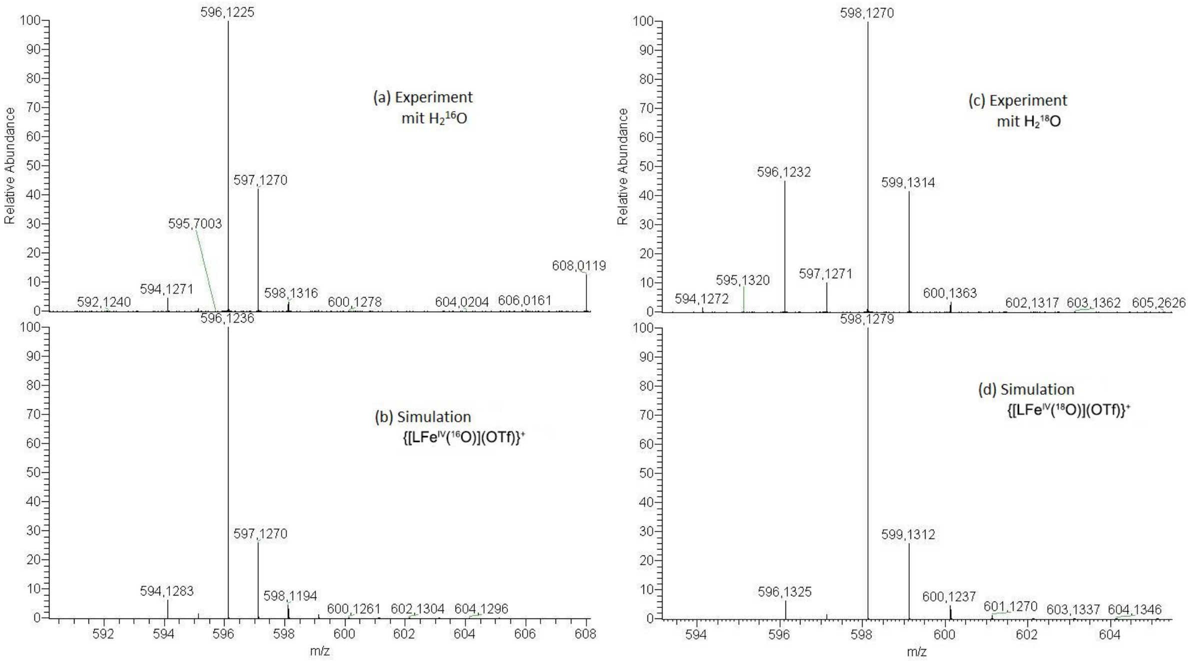

Fig. S3b: ESI-mass spectra of (a) unlabeled and (c) ^{18}O labeled reaction solutions of iron(II) precursor complex $[Fe^{II}(\mathbf{L})MeCN]^{2+}$ and PhIO in MeCN/water mixtures, respectively (isotopic labeling via addition of $^{16}OH_2$ and $^{18}OH_2$, respectively); (b) and (d) show simulations of the experimental data.

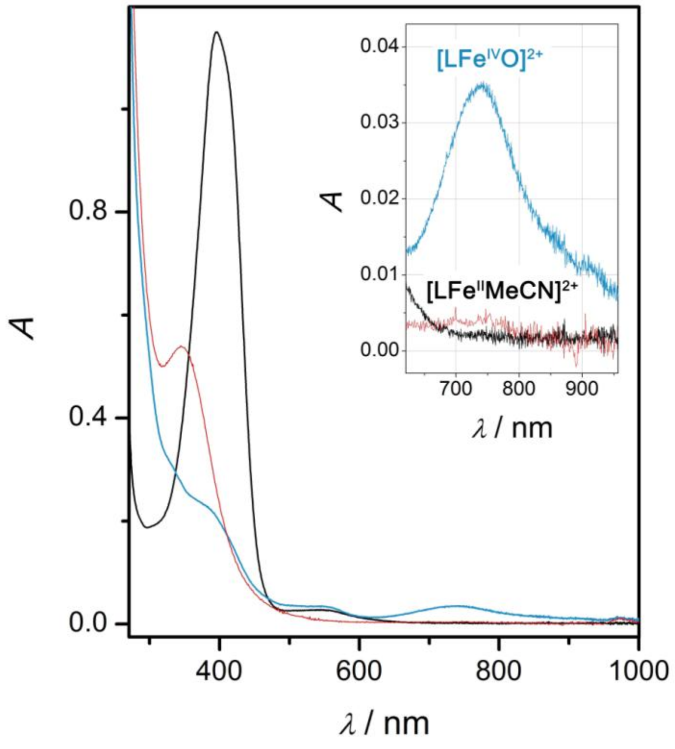

Fig. S3c: UV-Vis-NIR spectral dynamics of $[Fe^{II}(\mathbf{L})MeCN]^{2+}$ at RT (0.14 mM, MeCN, t = 0; black curve), 10 min after addition of 2 eq PhIO (blue curve) and after 10 h (red curve).

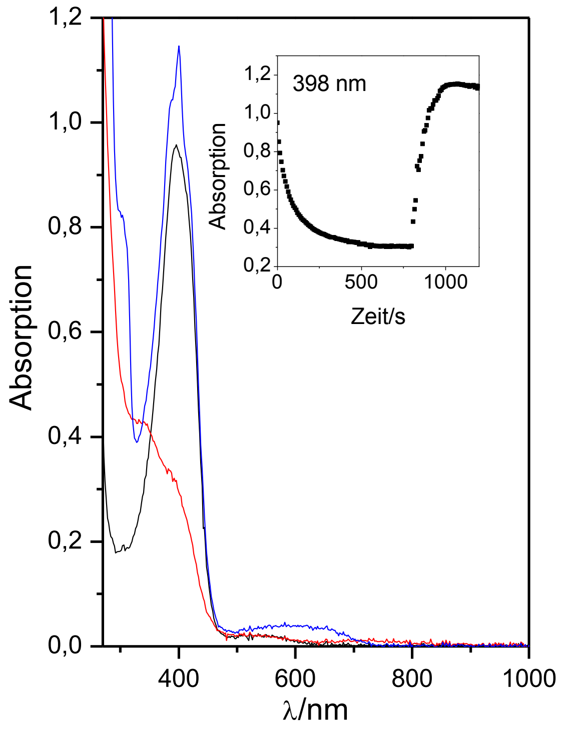

Fig. S4: Quenching of $[Fe^{IV}(\mathbf{L})(O)]^{2+}$ with 2,4,6-tris-^{tert}butyl-phenol in MeCN solution; black: $[Fe^{II}(\mathbf{L})MeCN]^{2+}$; red: + *m*CPBA (1 eq); blue: + PhOH (1 eq); inset: time profile of the absorption at λ = 398 nm; the quenching product is the 2,4,6-tris-^{tert}butyl-phenoxyl radical with diagnostic transitions at λ = 600 nm and λ = 400 nm.

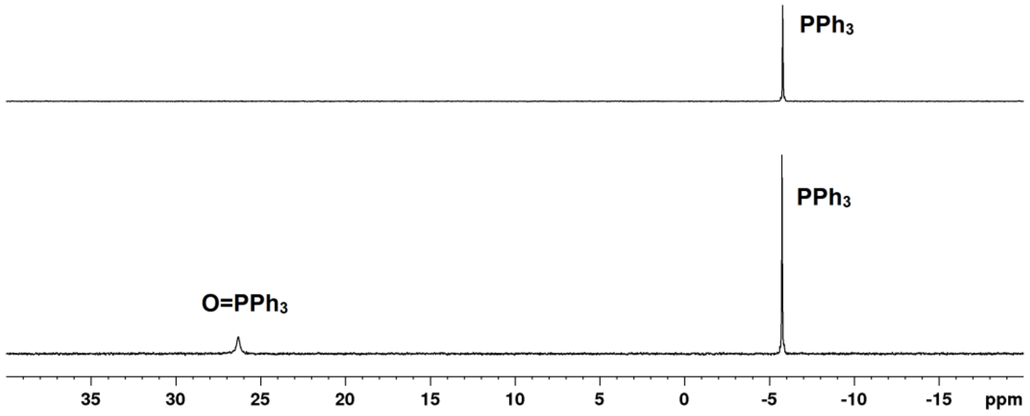

Fig. S5: Quenching of $[Fe^{IV}(\mathbf{L})(O)]^{2+}$ with triphenylphosphine in MeCN solution; top: ^{31}P NMR spectrum (81 MHz, d_3-MeCN, RT) of native PPh_3 in presence of PhIO; bottom: ^{31}P NMR spectrum (81 MHz, d_3-MeCN, RT) of $[Fe^{IV}(\mathbf{L})(O)]^{2+}$ after addition of PPh_3 (2 eq).

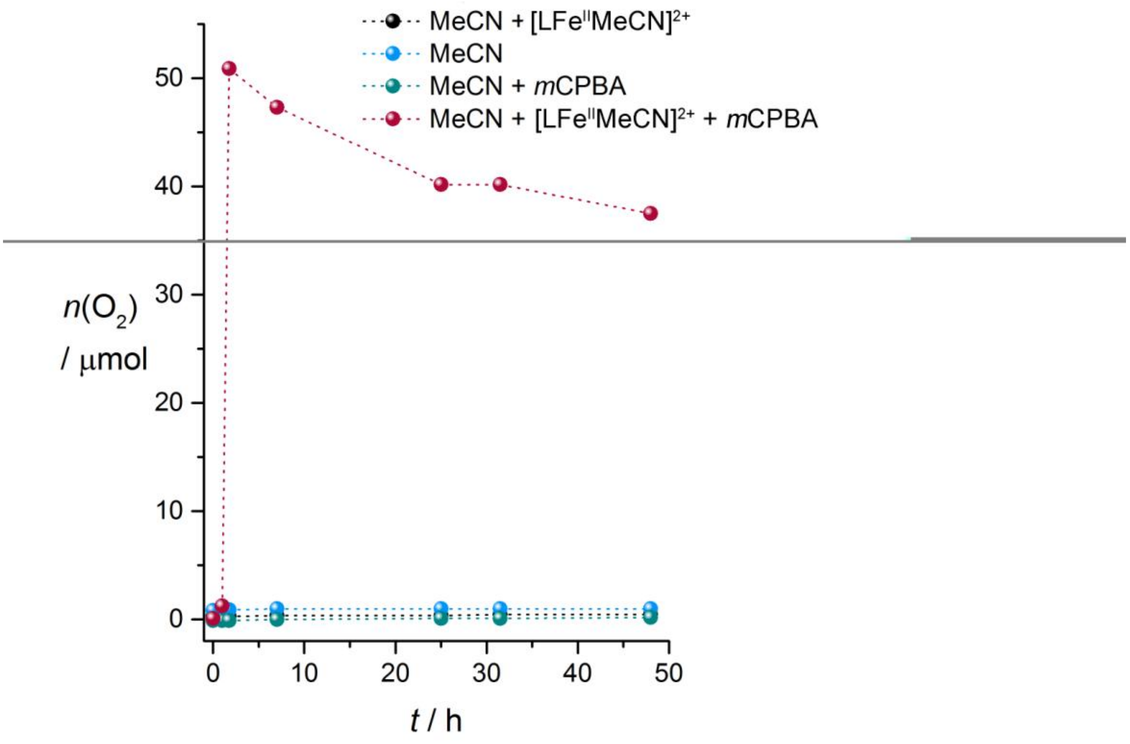

Fig. S6: Optometric head-space quantification of dioxygen after reaction of $[Fe^{II}(\mathbf{L})MeCN]^{2+}$ with excess *m*CPBA (10 eq) in MeCN; $[[Fe^{II}(\mathbf{L})MeCN]^{2+}]$ = 11 mM; V_{sol} = 0.01 L; $V_{headspace}$ = 0.02 L.

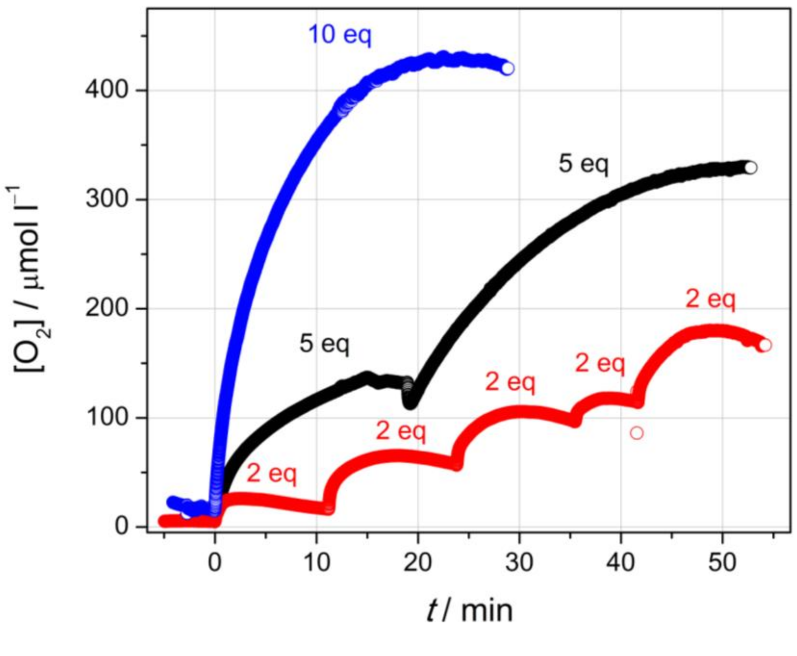

Fig. S7: Step-wise liberation of dioxygen from MeCN solution of $[Fe^{II}(\mathbf{L})MeCN]^{2+}$ after iterative addition of excess *m*CPBA.

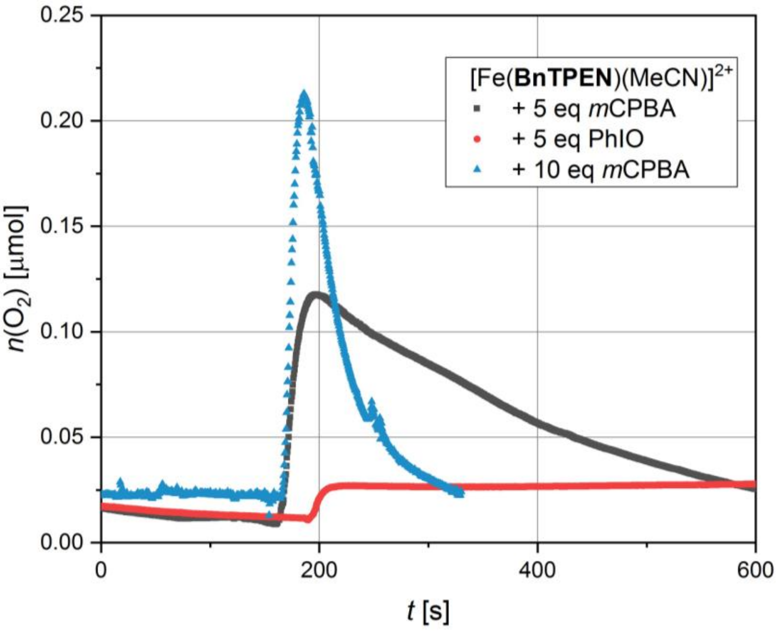

Fig. S8: Oxygen evolution (Clark-electrode system) from the oxoiron(IV) species $[Fe^{II}($**Bn-TPEN**$)(O)]^{2+}$, as synthesized in MeCN/water (1 : 4) from the reaction of $[Fe^{II}($**Bn-TPEN**$)(OTf)]^{+}$ with (a) 5 eq PhIO (red), (b) 5 eq *m*CPBA (black) and (c) 10 eq *m*CPBA (blue).

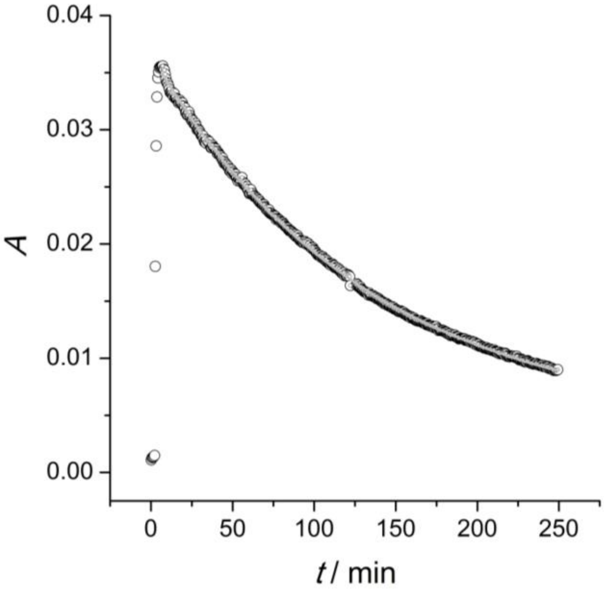

Fig. S9: Time evolution of the diagnostic NIR transition of $[Fe^{IV}($**L**$)(O)]^{2+}$ in MeCN (λ_{obs} = 730 nm); $[Fe^{IV}($**L**$)(O)]^{2+}$ formed in a reaction with PhIO at RT; its decay was recorded the absence of *m*CPBA; symbols: experimental data; line: mono-exponential fit to the data.

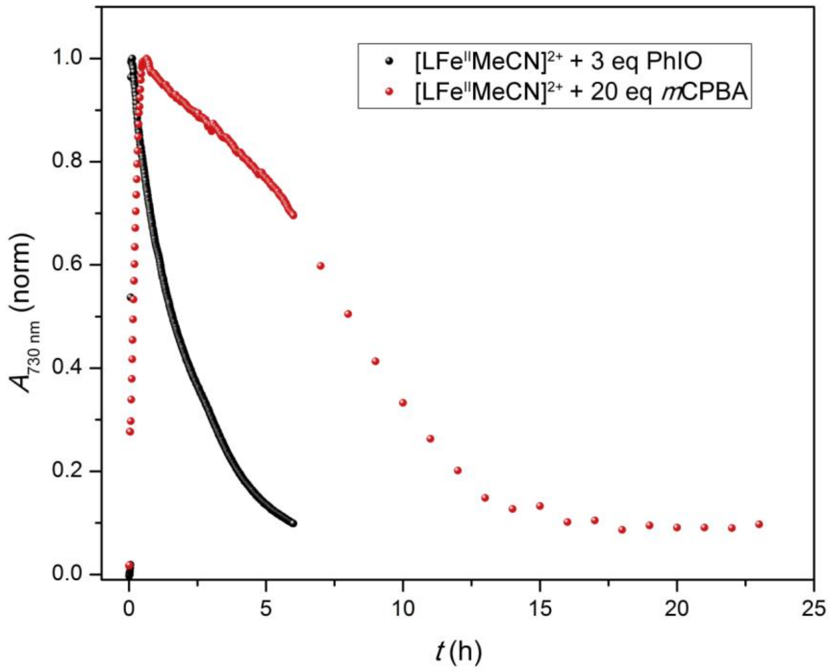

Fig. S10: Time evolution of the diagnostic NIR transition of $[Fe^{IV}(\mathbf{L})(O)]^{2+}$ in MeCN at RT (λ_{obs} = 730 nm); black: $[Fe^{IV}(\mathbf{L})(O)]^{2+}$ formed in a reaction with 3 eq PhIO; its decay was recorded the *absence* of *m*CPBA; red: $[Fe^{IV}(\mathbf{L})(O)]^{2+}$ formed in a reaction with 20 eq *m*CPBA.

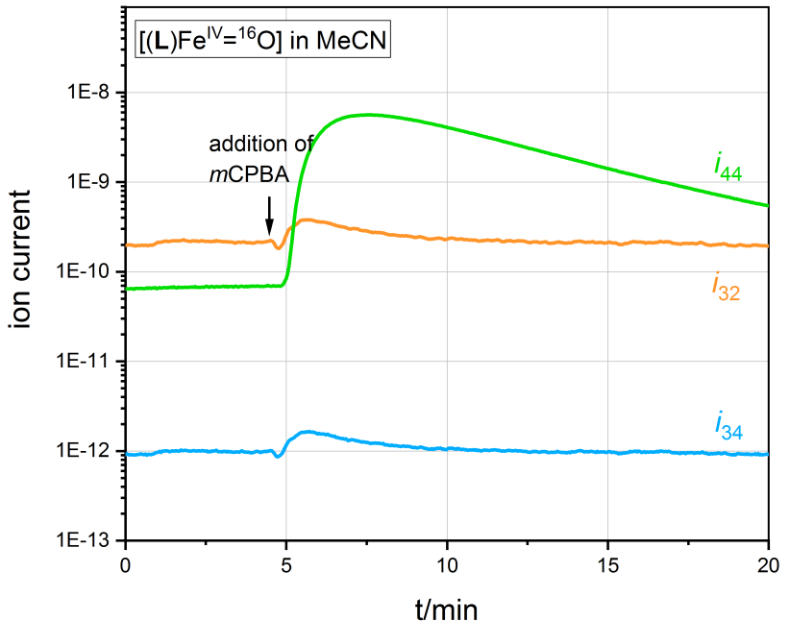

Fig. S11: In-situ MS ion currents of dioxygen isotopomers and carbon dioxide in "dry" MeCN solutions of $[Fe^{IV}(\mathbf{L})(O)]^{2+}$ (pre-synthesized via 10 mM $[Fe^{II}(\mathbf{L})(OTf)]^{+}$ + 2 eq PhIO) after addition of 10 eq *m*CPBA.

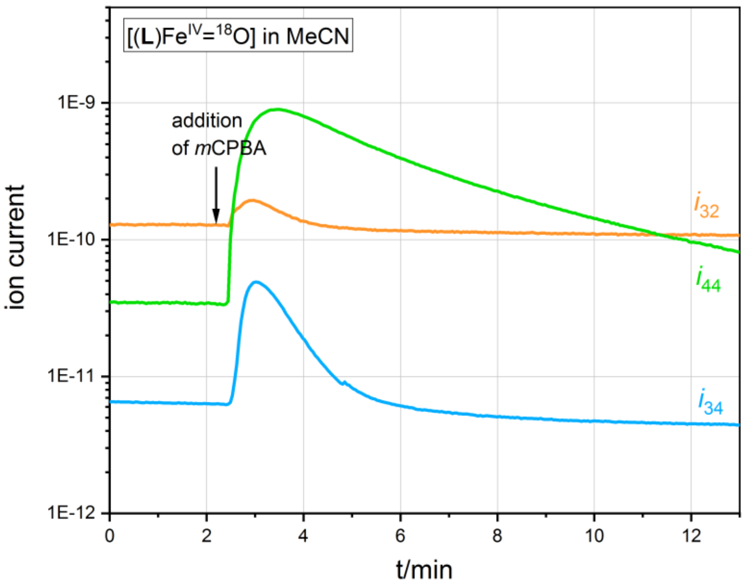

Fig. S12: In-situ MS ion currents of dioxygen isotopomers and carbon dioxide in MeCN solutions of $[Fe^{IV}(\mathbf{L})(O)]^{2+}$ (pre-synthesized via 10 mM $[Fe^{II}(\mathbf{L})(OTf)]^{+}$ + 2 eq PhIO) after labelling with 100 µl $^{18}OH_2$ for 30 min and addition of 10 eq *m*CPBA.

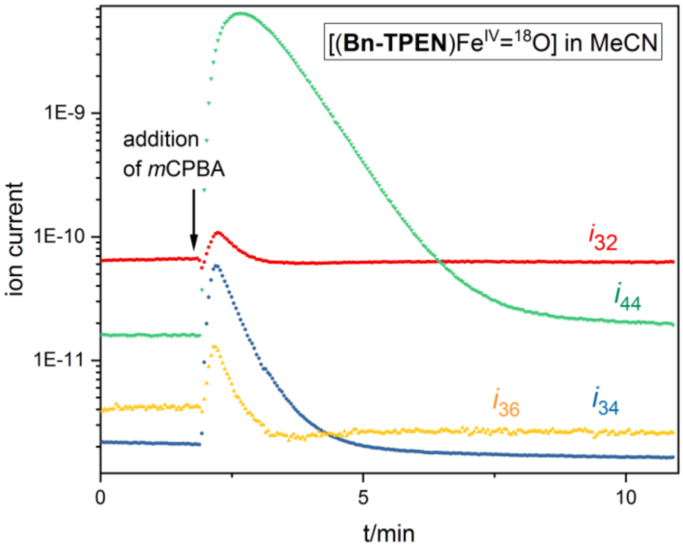

Fig. S13: In-situ MS ion currents of dioxygen isotopomers and carbon dioxide in MeCN solutions of $[Fe^{IV}(\mathbf{Bn\text{-}TPEN})(O)]^{2+}$ (pre-synthesized via 10 mM $[Fe^{II}(\mathbf{Bn\text{-}TPEN})(OTf)]^{+}$ + 2 eq PhIO) after labelling with 100 µl $^{18}OH_2$ for 30 min and addition of 10 eq *m*CPBA.

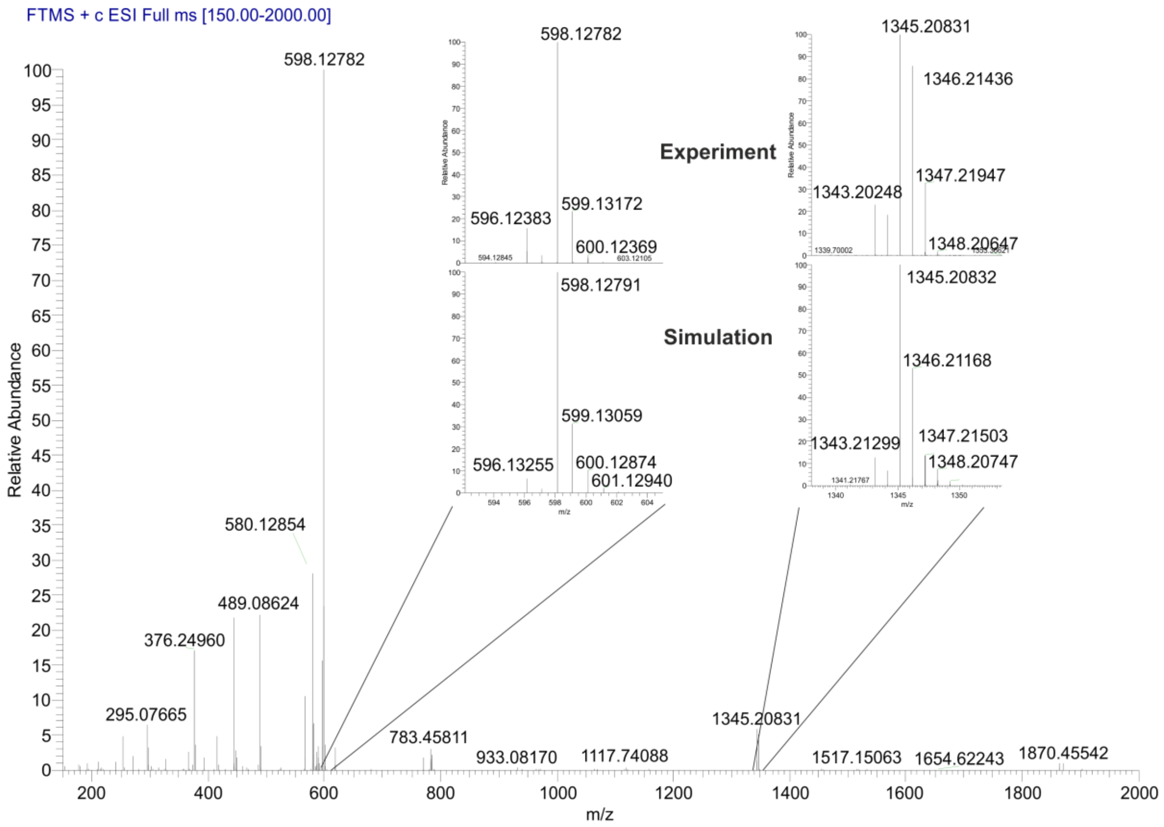

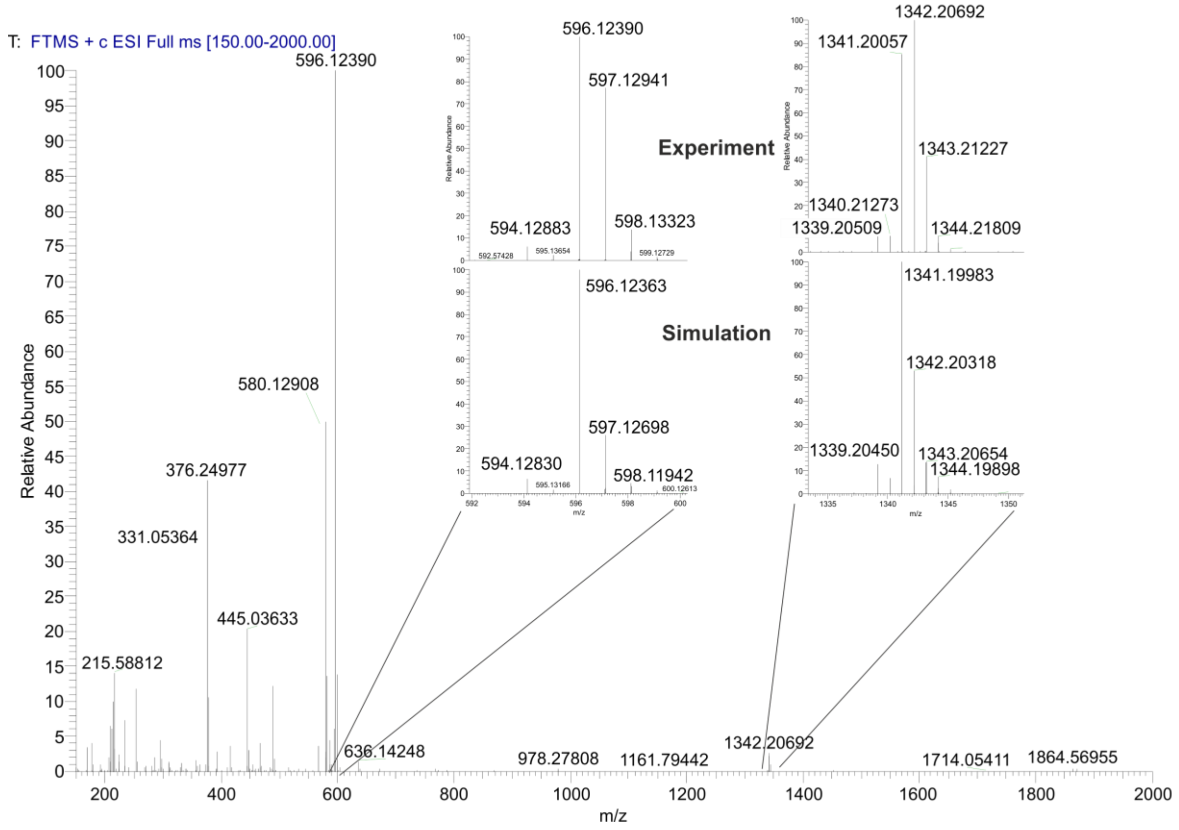

Fig. S14: Full-scan spectra of a) unlabeled and b) ^{18}O labeled reaction solutions of iron(II) precursor complex $[Fe^{II}$**(L)**$MeCN]^{2+}$ and *m*CPBA in MeCN/water mixtures, respectively (isotopic labeling via addition of $^{16}OH_2$ and $^{18}OH_2$, respectively). In the insets, experimental and simulated isotopic pattern of $\{[Fe($**L**$)(O)](OTf)\}^+/\{[Fe($**L**$)(^{18}O)](OTf)\}^+$ and $\{[Fe($**L**$)(O)]_2(OTf)_3\}^+/\{[Fe($**L**$)(^{18}O)]_2(OTf)_3\}^+$ are shown, respectively. Note the isobaric overlap of the dimers with the partially reduced complexes $\{[Fe($**L**$)(O)][Fe($**L**$)(OH)](OTf)_3\}^+$ and $\{[Fe($**L**$)(^{18}O)][Fe($**L**$)(^{18}OH)](OTf)_3\}^+$, respectively.

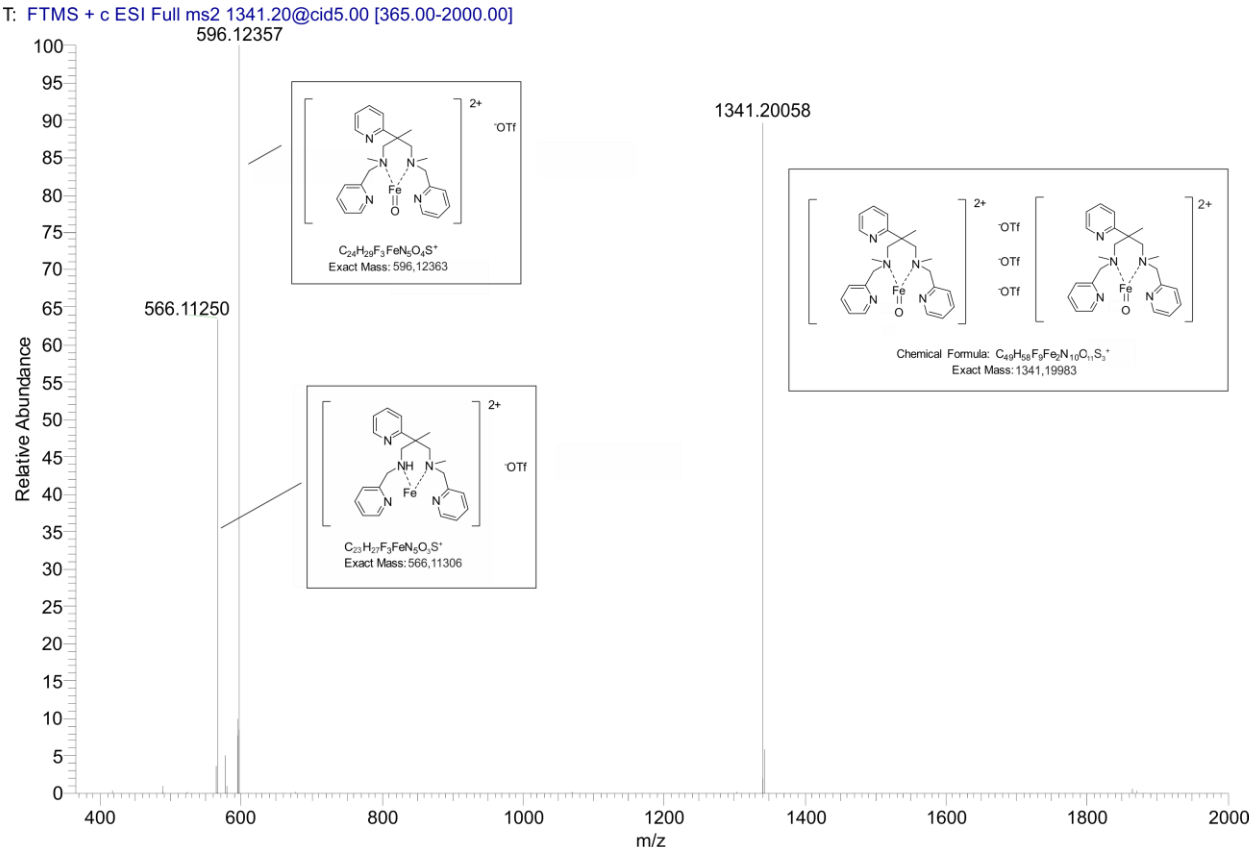

Fig. S15: Collision-induced dissociation of the triflate-bridged dimer {[Fe(**L**)(O)]$_2$(OTf)$_3$}$^+$ yielding the fragment ions {[Fe(**L**)(O)](OTf)}$^+$ and {[Fe(**L**–CH$_2$)](OTf)}$^+$.

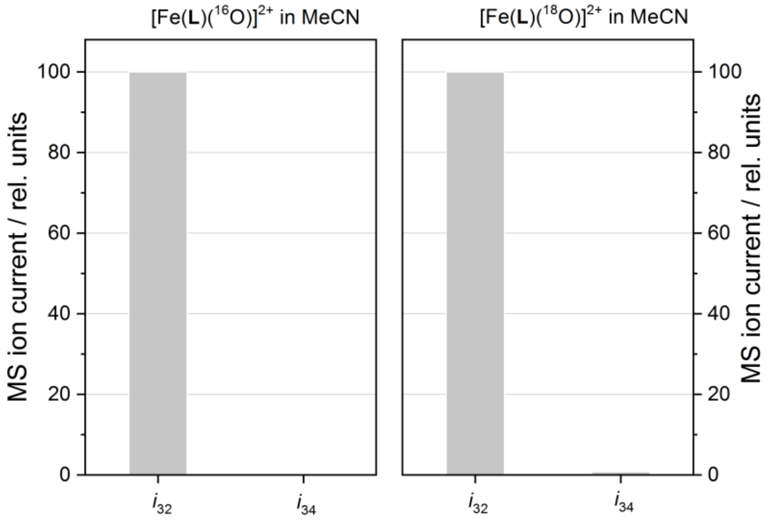

Fig. S16: In-situ MS analysis of dioxygen isotopomers in MeCN solutions of [Fe^{IV}(**L**)(O)]$^{2+}$ (pre-synthesized via 10 mM [Fe^{II}(**L**)(OTf)]$^+$ + 2 eq PhIO) after addition of 50 eq *t*-BuOOH (left) or after labelling with 100 µl ^{18}OH$_2$ for 30 min and addition of 50 eq *t*-BuOOH (right).

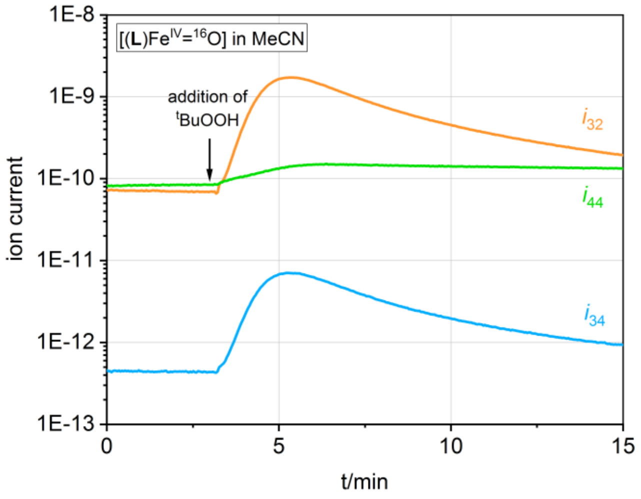

Fig. S17: In-situ MS ion currents of dioxygen isotopomers and carbon dioxide in MeCN solutions of $[Fe^{IV}(\mathbf{L})(O)]^{2+}$ (pre-synthesized via 10 mM $[Fe^{II}(\mathbf{L})(OTf)]^{+}$ + 2 eq PhIO) after addition of 50 eq *t*-BuOOH.

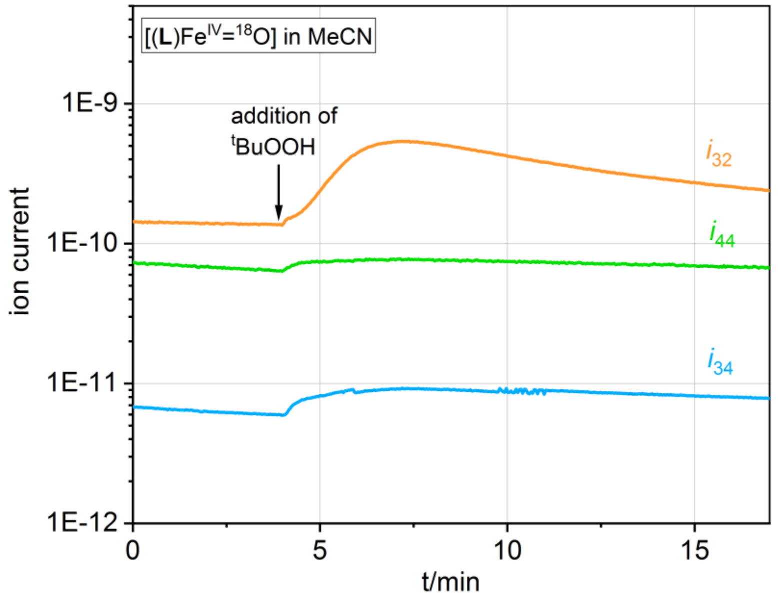

Fig. S18: In-situ MS ion currents of dioxygen isotopomers and carbon dioxide in MeCN solutions of $[Fe^{IV}(\mathbf{L})(O)]^{2+}$ (pre-synthesized via 10 mM $[Fe^{II}(\mathbf{L})(OTf)]^{+}$ + 2 eq PhIO) after labelling with 100 µl $^{18}OH_2$ for 30 min and addition of 50 eq *t*-BuOOH.

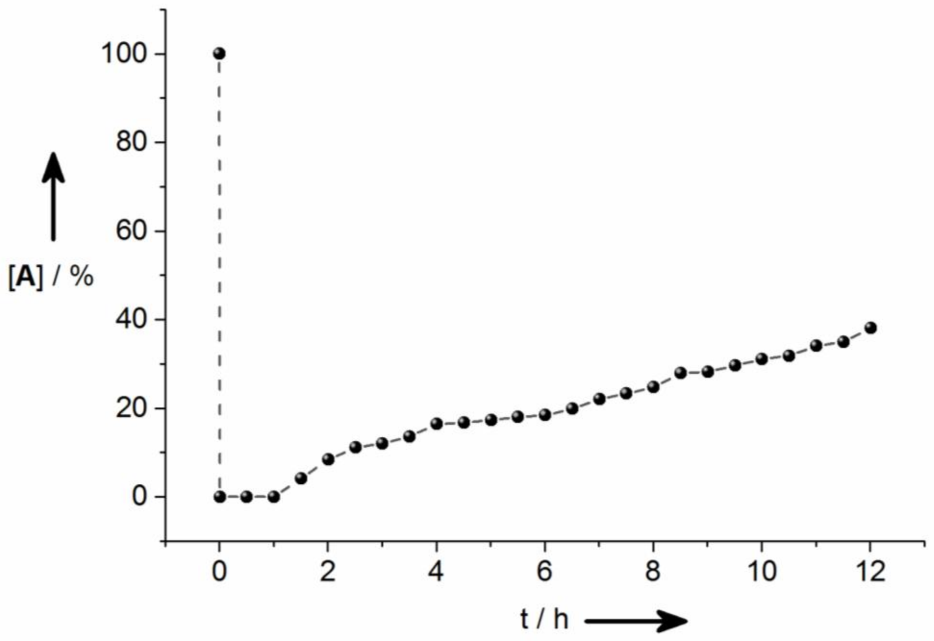

Fig. S19: ^{1}H-NMR recorded recovery of $[Fe^{II}(\mathbf{L})MeCN]^{2+}$ from aged mixtures of $[Fe^{II}(\mathbf{L})MeCN]^{2+}$ ($[[Fe^{II}(\mathbf{L})MeCN]^{2+}]_0$ = 20 mM) and *m*CPBA (10 eq) in d_3-MeCN at ambient temperature.
